# NNRTI and Liver Damage: Evidence of Their Association and the Mechanisms Involved

**DOI:** 10.3390/cells10071687

**Published:** 2021-07-04

**Authors:** Ana M. Benedicto, Isabel Fuster-Martínez, Joan Tosca, Juan V. Esplugues, Ana Blas-García, Nadezda Apostolova

**Affiliations:** 1 Department of Pharmacology, Faculty of Medicine, University of Valencia, 46010 Valencia, Spain; abe6@alumni.uv.es (A.M.B.); fusmari@alumni.uv.es (I.F.-M.); nadezda.apostolova@uv.es (N.A.); 2 Digestive Medicine Department, University Clinical Hospital of Valencia, 46010 Valencia, Spain; joantosca@gmail.com; 3 FISABIO–University Hospital Dr Peset, 46017 Valencia, Spain; ana.blas@uv.es; 4 Center for Biomedical Research Network–Hepatic and Digestive Diseases (CIBERehd), 46010 Valencia, Spain; 5 Department of Physiology, Faculty of Medicine, University of Valencia, 46010 Valencia, Spain

**Keywords:** antiretroviral drugs, HIV, hepatotoxicity, liver, DILI, cART

## Abstract

Due to the improved effectiveness and safety of combined antiretroviral therapy, human immunodeficiency virus (HIV) infection has become a manageable, chronic condition rather than a mortal disease. However, HIV patients are at increased risk of experiencing non-AIDS-defining illnesses, with liver-related injury standing out as one of the leading causes of death among these patients. In addition to more HIV-specific processes, such as antiretroviral drug-related toxicity and direct injury to the liver by the virus itself, its pathogenesis is related to conditions that are also common in the general population, such as alcoholic and non-alcoholic fatty liver disease, viral hepatitis, and ageing. Non-nucleoside reverse transcriptase inhibitors (NNRTIs) are essential components of combined anti-HIV treatment due to their unique antiviral activity, high specificity, and acceptable toxicity. While first-generation NNRTIs (nevirapine and efavirenz) have been related largely to liver toxicity, those belonging to the second generation (etravirine, rilpivirine and doravirine) seem to be generally safe for the liver. Indeed, there is preclinical evidence of rilpivirine being hepatoprotective in different models of liver injury, independently of the presence of HIV. The present study aims to review the mechanisms by which currently available anti-HIV drugs belonging to the NNRTI family may participate in the development of liver disease.

## 1. Introduction

**Figure 1:**
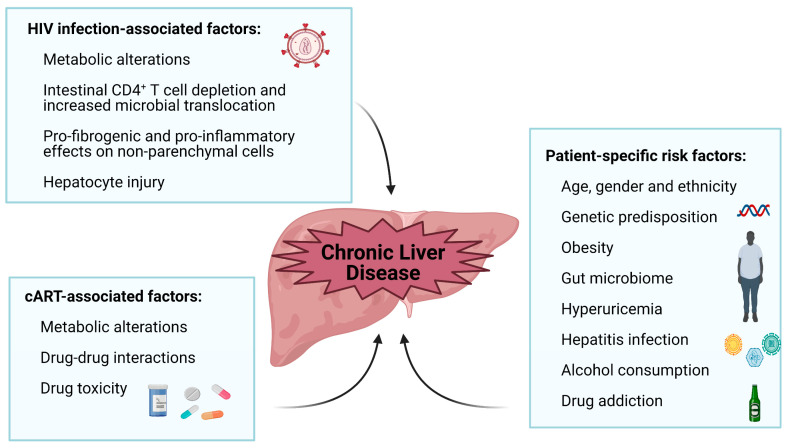
Factors that promote chronic liver disease in the HIV-infected population. These factors can be associated with HIV infection (direct effect of the virus on the liver that has been shown to produce metabolic alterations, increased microbial translocation, hepatocyte injury and pro-fibrogenic and pro-inflammatory effects on non-parenchymal cells) and with cART (ability of each ARV drug to induce hepatotoxicity and metabolic alterations). Furthermore, patients present specific risk factors (variations arising from patient’s sex, ethnicity and age, and other pathophysiological aspects such as alcohol consumption, obesity, viral hepatitis infection, genetic predisposition, hyperuricemia, and gut microbiome composition).

One of the greatest public health achievements of the past two decades was the advent of combined antiretroviral therapy (cART), which has dramatically diminished the incidence of opportunistic infections and the overall morbidity and mortality among patients with human immunodeficiency virus (HIV) infection. Despite these positive outcomes, HIV patients are at greater risk of developing non-AIDS conditions, with chronic liver disease (CLD) being among the most prominent [1,2]. Liver-related disease, including chronic viral hepatitis, is one of the leading causes of non-AIDS related deaths among patients, accounting for 13% according to the large D:A:D study (data from the period 1999–2011) [3], or as much as 21% of all-cause mortality in HIV patients in another study (some years during the period 1996–2004) [4]. Increased prevalence and earlier onset of liver disease in these patients is related to multiple factors, including the presence of the virus itself, immune-mediated injury, cytotoxicity, gut microbial translocation and systemic ongoing inflammation [1]. Additionally, cART is believed to play a major role, and this is related to the fact that the most common general complications associated with cumulative exposure to cART include the main risk factors for the CLD, namely metabolic changes (body fat redistribution, insulin resistance, diabetes mellitus and hyperlipidemia) and oxidative stress [5]. Moreover, hepatotoxicity or drug-induced liver injury (DILI) is one of the most common adverse reactions induced by antiretroviral (ARV) drugs. Considering that a significant portion of HIV patients are coinfected with hepatitis B/C virus, which has a cumulative impact on the liver, pre-existing liver disease is also regarded as a crucial factor for the development of liver toxicity in patients on cART. A decade ago, HIV/HCV coinfection was reported to lead to accelerated liver fibrosis (LF) and to higher rates of liver failure and death compared with HCV monoinfection [6]. More recently, with the availability of new directly acting antiviral agents (DAA), coinfected patients still display a higher mortality risk and more severe hepatocellular carcinoma compared to HCV monoinfected patients, despite the dramatically improved clinical outcome [7]. Among the many contributing factors, accumulated toxicity as a result of simultaneous cART-DAA treatment can also play a role. Nevertheless, even without hepatitis virus coinfection, HIV-1–infected patients are at a significant risk of LF [8]. In summary, hepatic pathogenesis in HIV patients is related to a complex combination of factors, as schematically shown in Figure 1.

**Figure 2:**
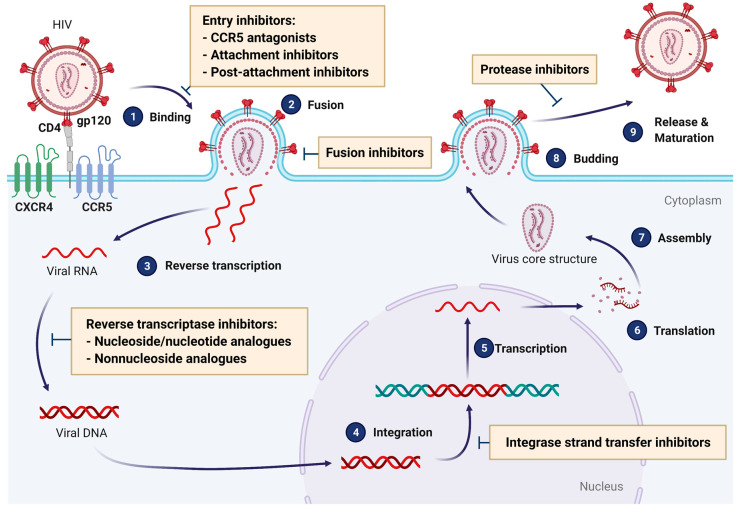
Available anti-HIV drug families and their mechanisms of action. Antiretroviral drugs include entry inhibitors, fusion inhibitors, nucleoside/nucleotide analogue reverse transcriptase inhibitors (N(t)RTIs), non-nucleoside reverse transcriptase inhibitors (NNRTIs), integrase inhibitors (INSTIs), and protease inhibitors (PIs). Entry inhibitors and fusion inhibitors prevent HIV from entering target cells by inhibiting its binding or its fusion, respectively. The exact mechanism involved varies depending on which receptor or complex formation is blocked: inhibition of CCR5 interaction with viral gp120 protein (CCR5 antagonists), blocking of viral gp120 (attachment inhibitors), or inhibition of the conformational changes of CD4/gp120 complex, which allow the binding to co-receptors (CCR5 or CCR4) (post-attachment inhibitors). N(t)RTIs, which can be either nucleoside or nucleotide analogues, function by inhibiting the synthesis of DNA by reverse transcriptase, the viral enzyme that copies viral RNA into DNA in newly infected cells, acting as false nucleotides. NNRTIs also inhibit the synthesis of viral DNA, but by binding to and inhibiting the reverse transcriptase in a non-competitive way. INSTIs inhibit the viral integrase, thus impeding the incorporation of reverse-transcribed HIV DNA into host cell DNA. Finally, PIs bind to the active site of the viral protease enzyme, preventing the processing of immature viral proteins into their functional conformations and the generation of new viral particles.

There are currently nearly 40 anti-HIV drugs belonging to six major classes, shown in Figure 2. Of the four enzymatic activities found in HIV-1 proteins (protease, reverse transcriptase polymerase, reverse transcriptase RNase H, and integrase), only RNase H has no approved therapeutical agents directed against it. Actually, many RHIs have been reported in the literature as effective inhibitors in enzyme assay; however, their high levels of toxicity or lack of selective inhibition have hampered their development for clinical use [9]. Combination therapy is still the gold standard for treatment of HIV infection, and regimens are tailored to the individual in order to minimize toxicity/intolerance and diminish the risk of developing resistance [10].

Non-nucleoside reverse transcriptase inhibitors (NNRTIs) are essential components of cART due to their unique antiviral activity, high specificity, and acceptable toxicity. So far, six NNRTIs have been approved by FDA or EMA for HIV-1 treatment; the first generation includes nevirapine (NVP, 1996), delavirdine (DLV, 1997, later discontinued) and efavirenz (EFV, 1998), and the second-generation compounds are etravirine (ETR, 2008), rilpivirine (RPV, 2011) and doravirine (DOR, 2018). The main pharmacological characteristics of these drugs are displayed in Table 1. In addition, elsulfavirine was approved only in Russia (ESV, 2017) and there are several new NNRTIs currently under development.

The present study aims to review the mechanisms by which currently available anti-HIV drugs belonging to the NNRTI family can cause liver injury. To this end, data obtained in vitro (cellular models) and in vivo (experimental animals) will be analyzed in light of evidence obtained from clinical trials.

## 2. Liver Damage: General Aspects and cART-Related Effects

Liver damage can take the form of three main patterns of injury: cholestatic, hepatocellular and mixed. It is clinically manifested by the presence of abnormalities in liver function tests, specifically elevated serum levels of alkaline phosphatase, aminotransferases and bilirubin. Severe hepatotoxicity is generally considered to be present if grade 3 or 4 transaminase elevations (TE) are detected in serum, which is 10 times (TE3) and > 10 times (TE4) the upper level of normality (ULN) for aspartate aminotransferase (AST) and/or alanine aminotransferase (ALT) levels for patients that have normal levels at baseline coinfection [16,17,18,19]. Chronic hepatocellular injury refers to prolonged damage to hepatocytes, as occurs in chronic viral hepatitis, alcoholic liver disease, DILI, and non-alcoholic fatty liver disease (NAFLD), while chronic cholestatic injury results from prolonged biliary obstruction, as seen in primary sclerosing cholangitis and primary biliary cholangitis [20]. There is controversy as to how DILI compromises the liver in the long term; however, it is known that protracted chronic injury, both hepatocellular and cholestatic, can lead to LF and ultimately liver architectural destruction and cirrhosis. In patients who have experienced an episode of severe DILI, clinically relevant liver disease rarely ensues; however, 1% of these patients will go on to develop cryptogenic cirrhosis (mainly those who initially present with hepatocellular type DILI). Continuing treatment with a drug that has begun to cause liver injury may increase the risk, not only of more severe acute damage, but also chronic injury.

Hepatocytes, which represent 80% of the total liver cell number, have traditionally been studied as the primary target of toxic insults in the liver. Not only hepatocytes, but also non-parenchymal cells, such as hepatic stellate cells, cholangiocytes, Kupffer cells, and sinusoidal endothelial cells, can be implicated in the process of cART-induced hepatotoxicity; however, studies that analyze the effects on non-parenchymal cells are scarce. One in vitro study that explored the actions of the anti-HIV drug efavirenz and the combination of lopinavir+ritonavir on primary mouse hepatocytes, Kupffer cells and hepatic stellate cells revealed differential effects regarding cytotoxicity and ER stress on parenchymal versus non-parenchymal cells [21]. In addition, in macrophages, HIV protease inhibitor lopinavir-induced TNF-alpha and IL-6 expression is coupled to the unfolded protein response and ERK signaling pathways [22]. It is of note that cART has been associated with vanishing bile duct syndrome (VBDS), a group of rare disorders characterized by ductopenia, progressive destruction and disappearance of intrahepatic bile ducts leading to cholestasis [23]. The pathogenic mechanism of VBDS is still elusive and therefore how antiretroviral drugs contribute to it is still not understood.

Liver toxicity caused by cART is believed to be inflicted through several mechanisms which can be categorized as hypersensitivity reactions (idiosyncratic hepatotoxicity), direct mitochondrial inhibition, direct cell stress, or immune reconstitution, particularly in the presence of viral hepatitis coinfection [24], while some authors have also identified disturbances of lipid/sugar metabolism and steatosis as a separate mechanism [25]. In fact, lipids, with their diverse metabolic, inflammatory, and immune response functions, are intimately linked to the pathology of CLD, particularly NAFLD.

Various antiretroviral drug families have been shown to induce hepatic effects to a different degree. The reader is referred to several recent comprehensive reviews on the subjects [26,27,28]. In the case of NRTIs, moderate to severe hepatotoxicity rates have been reported for zidovudine, stavudine and didanosine while the newer drugs, emtricitabine, tenofovir, abacavir and lamivudine are associated with a significantly lower risk. The mechanism of NRTI-induced hepatic injury particularly in the case of the first-generation-NRTIs is related to their capacity to produce mitochondrial toxicity, a distinctive DILI pattern causing acute liver failure preceded by tender hepatomegaly (due to steatosis) and lactic acidosis [29]. Some of the manifestations are genetically determined, such as the case of abacavir-induced hypersensitivity, which has greater incidence in individuals with HLA-B*5701 phenotype [30]. As a group, protease inhibitors also show moderate to high rates of hepatotoxicity and elevations in serum ALT/AST >5 times ULN are reported in up to 15% of the patients and are more common in patients with HIV-HCV coinfection [29]. While immuno-allergic features are rare, atazanavir and indinavir enhance unconjugated serum bilirubin due to the inhibition of UDP glucuronyl-transferase in a Gilbert syndrome–like fashion with jaundice not indicative of hepatic injury [29]. Hepatotoxicity induced by entry inhibitors has also been reported. Case reports of maraviroc-related DILI exist [31,32] and in 2005, the development of aplaviroc, another CCR5 antagonist, was halted because of severe hepatotoxicity [33]. Finally, INSTIs, the current class of choice in first-line regimens seem to be rather hepatic-safe [34]. Notably, neither elevated alkaline phosphatase nor ALT level were reported among patients in treatment-naïve HIV patients on dolutegravir containing therapy for 96 weeks [35]. In a review of the incidence of hepatotoxicity with INSTIs use in 4366 people participating in The EuroSIDA study, a prospective observational pan-European cohort study of people living with HIV-1 across Europe, there was only one discontinuation due to hepatotoxicity [36].

Importantly, due to the fact that antiretroviral drugs are given as a combination therapy, the capacity of different anti-HIV drug families and that of specific drugs to exert cumulative hepatotoxic effects need to be considered. In this regard, there are case reports for developing severe liver injury with combination of drugs that are not particularly considered hepatotoxic, such as the combination abacavir/lamivudine/dolutegravir in HLA-B5701-negative patients [37,38].

## 3. Mechanisms of NNRTI-Induced Liver Injury

A review of the literature clearly shows that first-generation NNRTIs exert significant hepatotoxicity, unlike their second-generation counterparts, which seem to be safe for the liver, even in patients coinfected with hepatitis viruses. It is of note that there are various reports of a lack of association of the presence of advanced LF or cirrhosis with an increased risk of grade 3 or 4 TE in HIV patients exposed to second-generation NNRTIs [39,40]. The lower incidence and severity of hepatic toxicity reported with second-generation NNRTIs is related to the rare occurrence of hypersensitivity reactions and the lower potential for induction of liver enzymes exerted by these drugs.

### 3.1. Nevirapine

Nevirapine (NVP) is not currently recommended in initial anti-HIV regimens [41,42]. However, it continues to be used in a certain number of HIV-1 individuals due to its high efficacy, good metabolic profile, convenience, and low cost, particularly as part of cART simplification strategies [43,44,45]. Hepatotoxicity has been reported more commonly with NVP than with other ARV drugs [46,47,48], sometimes exceeding that induced by the other first-generation NNRTI, EFV, and requiring discontinuation more frequently [49,50,51,52,53,54]. In fact, in the year 2000, the FDA announced a black box warning on NVP due to hepatotoxicity. Liver injury occurs in 6–17% of patients under therapy with NVP [50,55], while the incidence of severe liver toxicity has been reported in the range of 1.3–12% [51,55,56,57].

A significant number of individuals exposed to NVP develop early and short-term hypersensitivity reactions (usually occurring within 6–12 weeks of administration), which can manifest as hepatotoxicity and/or cutaneous adverse reactions ranging from rash to serious skin reactions [49,58]. These effects seem to be immune-mediated and are not related to plasma concentration of NVP [58]. Late-onset hepatotoxicity commonly starts 4–5 months after initiation of NVP treatment, and its underlying mechanism is less understood. Thus, reported NVP-induced hepatotoxicity comprises several clinical manifestations with varying degrees of severity including elevation of serum enzyme levels, bile duct obstruction and jaundice, hepatic necrosis, hepatitis and hepatic failure [49,51,58,59,60,61,62]. NVP’s associated hepatotoxicity in the clinics (case reports, clinical trials and cohorts’ studies) has been reviewed in detail elsewhere [63]. A structured review of the literature (2005–2015) also revealed that NVP causes hepatotoxicity in pregnant patients, manifested as elevation of transaminases, rash, hepatitis, pruritus, abdominal pain, eosinophilia, encephalopathy, and jaundice [64]. The link between NVP and more advanced stages of liver disease has also been assessed through studies with HCV-coinfected patients, obtaining somewhat contradictory results. While in one study NVP use was associated with severe LF (unrelated to the duration of the exposure [65]), other authors suggested that NVP was associated with a low probability of significant LF [66] or was even found to be protective against LF progression [67].

The mechanisms of hepatotoxicity induced by NVP have been suggested by several in vivo studies. At a dose of 150 mg/kg/day administered in a chow diet during a 5-week period, NVP-treated female Sprague Dawley rats developed a rash and displayed hepatic alterations (larger livers and inclusions observed in hepatocytes in the centrolobular region, which may be related to NVP’s inducing of P450 enzymes in the endoplasmic reticulum (ER)) [68]. However, the authors showed that the effects on the liver did not resemble NVP-induced liver toxicity in humans and occurred in the absence of any increase in transaminases. Another study reported that NVP (200  mg/kg/day p.o) caused subclinical liver injury (with no elevations in the plasmatic levels of liver enzymes) in rats within hours after the first dose, which continued for up to 7 days but was not detected at 14 or 21 days of treatment. In the first 24 h of treatment, liver injury manifested as degenerative changes with a granular appearance and vacuolar degeneration of hepatocytes, increased apoptosis, and dissociated liver parenchyma. The authors suggested that NVP acts as an immune stimulator, more specifically due to the generation of toxic metabolites [69]. The aberrant immune response to the genesis of idiosyncratic drug reactions in general is thought to arise as a result of covalent binding of drugs to proteins in the affected tissues. In line with this, one study showed hepatic covalent binding of NVP and suggested that the quinone methide metabolite was responsible for NVP-induced liver injury [70]. The same study reported evidence of mild, delayed-onset liver injury in C57BL/6 mice exposed to NVP for 3 weeks (950 mg/kg/day in standard chow diet), which presented as hepatocyte death in some lobular areas, while in some animals there was only a small increase in ALT that resolved itself despite continued treatment. NVP is metabolized by cytochrome P450, resulting in four mono-oxygenated metabolites, of which one, 12-hydroxy-NVP (12-OH-NVP), a major participant in the development of rash, may also contribute to hepatic toxicity, though to a lesser extent than the parent drug [70]. The same group also studied gene expression in the livers of female Brown Norway rats after 6 and 12 h treatment with NVP or 12-OH-NVP via gavage (equimolar dosages of 150 or 159 mg/kg/day respectively) [71]. Both NVP and 12-OH-NVP induced changes in hepatic gene expression, but the list of affected genes varied, thus pointing to different bioactivation pathways. Most of the up-regulated genes were involved in drug metabolism, protein folding and immunity, while, interestingly, the most down-regulated gene was neuronal regeneration-related protein (Nrep), also known as P311, a protein whose diminished levels have recently been correlated with NAFLD [72]. Nrep down-regulation activates the TGF-β receptor/PI3K/protein kinase B pathway, thus stimulating hepatic acetyl-CoA and cholesterol synthesis. Whether Nrep has anything to do with the hepatic or metabolic effects of NVP in humans is yet to be determined.

Brown Norway rats treated with NVP (150 mg/kg/day for 8 weeks, through diet) showed a heterogeneous pattern of liver damage, by which some hepatocytes were affected by the drug and developed necrotic cytoplasm and mitochondrial degeneration, while others appeared to be unaffected. Hepatocyte injury was demonstrated by the presence of granular cytoplasm, abnormal lipid inclusions, prevalent destruction of mitochondrial inner membrane and lipid-smooth endoplasmic reticulum (LSER) inclusions, and liver biopsies from treated rats also displayed varying degrees of endothelial abnormalities [73]. Hepatocytes do not seem to further process LSERs, but rather to expel them into the blood stream, from where they can be picked up by lymph nodes and contribute to initiation of an immune response that leads to serious liver injury or skin rash. In NVP-administered rats, the formation of LSER inclusions appears to be a mechanism of sequestration and evacuation of this NNRTI or its metabolites from hepatocytes abnormalities [73]. Although NVP causes an immune-mediated skin rash in Brown Norway rats, it does not cause significant hepatic necrosis as measured by serum ALT, presumably because the dominant immune response in the liver is immune tolerance. However, a significant increase in ALT upon NVP treatment (~950 mg/kg/day, 3 weeks, through diet) was indeed observed in mice in which PD1 and CDLA-4, two important immune checkpoints, were inhibited [74]. This hepatic injury was reversible, as ALT levels showed a tendency toward normalization after the third week of treatment.

Multiple in vitro studies have evaluated the effect of NVP in different types of cells. It is important to stress that NVP is metabolized though CYP enzymes, whereas certain cell lines, such as the human hepatoma HepG2, contain low levels of phase I and phase II enzymes. Therefore, studies in this cellular background provide information only about the direct effect of NVP on liver cells, and not that of its metabolites. NVP, albeit at concentrations higher than average plasma concentrations in patients, has been shown to inhibit proliferation of both HepG2 and THLE2 cells (human immortalized hepatocytes) to a similar degree, leading to necrotic and not apoptotic cell death [75]. The highest concentration used, or prolonged treatment with NVP, produced G2/M and G1/G0 phase cell cycle arrest in HepG2 and THLE2 cells, respectively. In addition, it induced cellular senescence in THLE2 cells, but not in hepatoma cells. IC50 for NVP in HepG2 after 24 h treatment was calculated as 819 μM, and the study revealed that NVP was pro-apoptotic. Long term (6-week) treatment of these cells showed that concentrations up to 273 μM did not affect cell viability [76]. The same group also reported data obtained from a proteomics study performed with HepG2 cells cultured for one week with 819 μM NVP, in which nearly 40% of the differentially expressed proteins were mitochondrial proteins, providing evidence of NVP interfering with mitochondria in this model [77]. Conversely, we have shown that NVP lacks mitochondrial toxicity in another human hepatoma cell line, Hep3B, though the treatment in question (10–50 µM) lasted only a few hours [78]. Differences in the expression of drug-metabolizing genes between HepG2 and Hep3B cells may explain this discrepancy.

In a recent study using primary mouse hepatocytes, supra-therapeutic concentrations of NVP induced cell death, while a version of the drug in which the 12th position had been trideuterated (12-D3NVP) exerted a lesser effect than NVP [79]. In another study, two-day exposure of primary hepatocytes to supratherapeutic concentrations of NVP in a flow-based culture system led to inhibition of EIF2 signaling, and was predicted to suppress downstream ATF4- and CHOP-related apoptotic pathways (revealed by analysis of transcriptomic data), as well as PDI and heat shock responses [80]. The same study showed that fatty acid synthesis and mitochondrial fatty acid β-oxidation were not affected by NVP; however, pathway analysis based on gene expression revealed increased mitochondrial respiratory activity by up-regulating the activities of complexes I–IV of the electron transport chain.

Unlike other NNRTIs that seem to be potent activators of key ligand-activated transcription factors in vitro, the effect of NVP is less clear. According to some studies, it does not seem to activate the pregnane-X receptor (PXR) [81,82], while evidence regarding the effect on the constitutive androstane receptor (CAR) is ambiguous [81,83]. An in vitro study that employed primary human hepatocytes within HemoShear devices which allow for the control of hemodynamics and transport revealed that treatment with NVP (48 h) produces a pattern of gene expression that is predictive of the activation of CAR-RXR signaling [80]. CAR and PXR not only act as sensors of drugs and xenobiotic substances that up-regulate the expression of metabolizing—such as cytochrome P450, phase II enzymes and transporters responsible for metabolism and excretion—but they also regulate the metabolism of endobiotics that may be relevant for NNRTI-induced metabolic alterations. The same study also suggested that NVP up-regulates pathways involved in bile acid synthesis, thus pointing to the cholestatic potential of NVP due to increased bile acid synthesis via the classic, CYP7A1-mediated pathway, rather than transport inhibition through the up-regulation of efflux bile acid transporters [80]. This effect may explain the clinical potential of NVP to promote cholestasis. Lastly, unbiased pathway analysis carried out by the same authors suggested that NVP regulates the antigen presentation pathway and predicts an increase of the expression of both major histocompatibility complexes (MHCs) class I and II, surface molecules that bind antigens and display them for recognition by T cells that mediate immunological responses. MHC class I are expressed on all nucleated cells, while MHC class II are expressed on antigen-presenting cells (APC), and to a lesser extent on hepatocytes. The immunogenic potential of NVP is supported by the finding that incubation of hepatocytes (FLC-4 cell line) led to the release of danger-associated molecular pattern molecules (DAMPs), which activate inflammasomes in macrophages derived from the THP-1 cell line [84]. Thus, inflammasome activation may be an important step in the activation of the immune system by drugs such as NVP.

**Figure 3:**
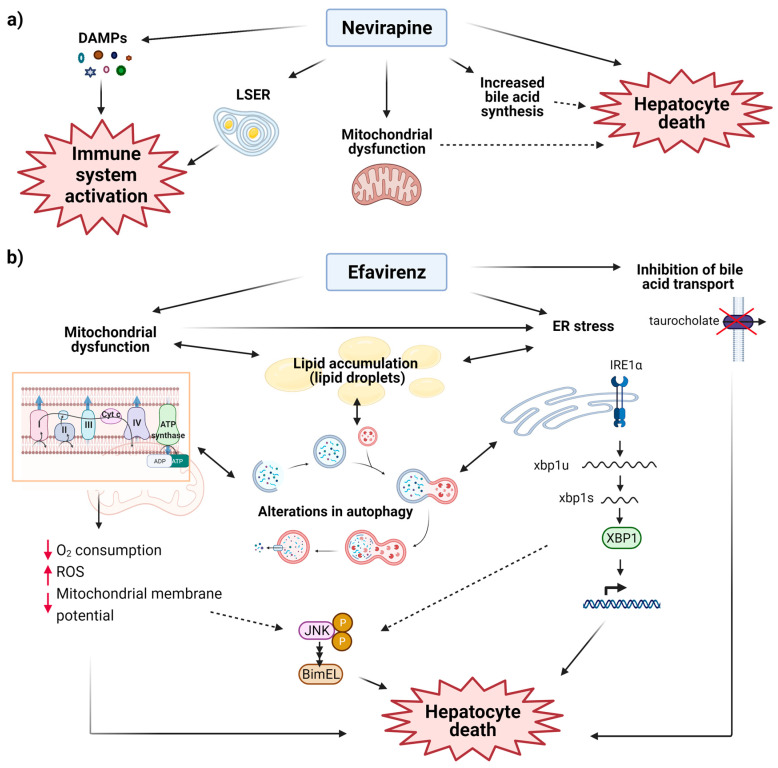
Mechanisms of NNRTI-induced liver toxicity suggested by in vitro and in vivo studies for (**a**) Nevirapine (NVP) and (**b**) Efavirenz (EFV). NVP leads to the release of danger-associated molecular pattern molecules (DAMPs) by hepatocytes, and to the formation of hepatocytic inclusions composed of lipid droplets surrounded by smooth endoplasmatic reticulum cisternae (LSER), which are deposited in sinusoids. These two events presumably contribute to triggering the immune response. NVP is also proposed to induce mitochondrial dysfunction and to increase bile acid synthesis, while at supra-therapeutic concentrations, this drug induces hepatocyte apoptosis. EFV inhibits complex I of the electron transport chain, which diminishes oxygen consumption and mitochondrial membrane potential, and increases reactive oxygen species (ROS) production, resulting in mitochondrial dysfunction. Moreover, this drug produces ER stress, since it activates inositol-requiring 1α (IRE1α) and, consequently, IRE1α-catalyzed splicing of X-box binding protein 1(XBP1) mRNA. EFV-induced mitochondrial dysfunction and ER stress are thought to be interconnected, to alter autophagic flux and to promote lipid accumulation inside hepatocytes. c-Jun N-terminal kinase (JNK) pathway is also activated in response to EFV, possibly related to alterations in mitochondrial and ER function. In addition, EFV also inhibits bile acid transport. All these events have been shown to promote hepatocyte death.

In conclusion, NVP produces idiosyncratic DILI (IDILI), whose mechanism is not fully understood, but seems to be related to the production of toxic and immunogenic metabolites in the liver. However, whether this effect accounts for the long-term hepatotoxicity of NVP is as yet unknown. A schematic representation of the mechanisms suggested to underlie NVP’s hepatotoxicity is provided in Figure 3A.

### 3.2. Efavirenz (EFV)

EFV was the NNRTI par excellence for 2 decades due to its inclusion as part of several successful first-line therapeutic regimens, including single-pill combinations with other anti-HIV agents. Nevertheless, this predominant position of EFV within initial cART regimens has been challenged by the arrival of newer antivirals with a better toxicological profile; EFV is no longer recommended for initial treatments by most of the international guidelines, except for certain clinical circumstances due to its efficacy, tolerability, and availability regimens [41,42]. On the negative side, EFV has been associated with several adverse events, including some effecting the liver. Severe increases (GE 3 or 4) in the plasma content of hepatic enzymes have been described in up to 48% of patients exposed to EFV [56,85,86]. This has been attributed to hypersensitivity reactions [87,88], reported very rarely, or to extensive accumulation of the drug inside the liver [59], which has a later onset (from 2 to 3 months onwards) and affects a large proportion of EFV-exposed patients suffering hepatic effects. Cases of acute liver failure with EFV, on the other hand, are rare. EFV has also been associated with alterations in bile acid metabolism, such as those reported by a case-control study in which an increase in the amount of total and direct bilirubin was observed after 24 months of EFV therapy, though figures did not exceed the clinical range of bilirubin [89]. This effect may be related to reports in human and rat hepatocytes of EFV inhibiting bile acid transport [90]. Importantly, there is abundant evidence that endorses the capacity of EFV to induce disturbances in the lipid metabolism of HIV patients, including effects on the liver. Notably, switching from EFV to other ARV drugs with better metabolic profiles has been shown to improve hepatic lipid metabolism. For example, HIV-infected individuals with NAFLD who switched from EFV to an integrase inhibitor showed decreases in the degree of hepatic steatosis compared with those who continued with EFV [91].

Several in vivo studies reported the capacity of EFV to alter liver metabolism and function [92], in line with what has been recorded in clinics. Namely, treatment of WT C57BL/6 mice with EFV (50–100 mg/kg/day for 1 week) produced hypercholesterolemia (but with unchanged plasma triglyceride levels) and hepatomegaly with liver steatosis [92].

Abundant evidence obtained in vitro with hepatic cells (hepatoma cell lines and primary hepatocytes) indicates that short-term treatments with clinically relevant concentrations of EFV compromise cell viability and alter mitochondrial function, diminishing mitochondrial transmembrane potential and increasing ROS generation [79,82,93,94,95,96]. We and others have reported an EFV-induced decrease in mitochondrial O_2_ consumption in cultured hepatic cells due to an inhibitory interference of EFV with complex I of the electron transport chain [79,95,97]. In addition, incubation of primary human hepatocytes with synthetic 8-hydroxyEFV (8-OHEFV), the primary metabolite of EFV, has been shown to result in cell death, caspase 3 activation and ROS formation [94]. This metabolite proved to be a more potent stimulator of cell death than EFV, and both EFV and 8-OHEFV mediated cell death through c-Jun *N*-terminal kinase (JNK) and Bcl-2 interacting mediator of cell death (Bim). JNKs are a family of serine/threonine kinases that are important regulators of cellular responses to stress, including cell death. There is also evidence that points to the capacity of EFV to induce ER stress and unfolded protein response (UPR) in human hepatic cells [98,99]. It activates key ER stress/UPR controllers, inositol-requiring 1α (IRE1α) and X-box binding protein 1 (XBP1) through increased formation of ROS, and this effect is thought to contribute to EFV-mediated hepatocyte death [99]. Our research shows that, concomitantly with mitochondrial dysfunction and ER stress, EFV-exposed cells display altered autophagy [100]. This adaptive and highly regulated process enables selective degradation of intracellular components as part of the cell’s response to a variety of stress stimuli (nutrient and energetic deprivation, altered proteostasis, etc.). While autophagy was found to be protective when triggered with lower concentrations of EFV, cytotoxic EFV levels were associated with a blockage of autophagic flux. Moreover, human hepatocytes cultured in the presence of EFV accumulate neutral lipids [79], which is relevant in the light of the enhanced hepatic steatosis reported in patients treated with this NNRTI.

Activation of nuclear receptors is another mechanism that may be involved in the side effects of EFV. In cultured hepatic cells, EFV activates the nuclear receptors PXR [82,92,101,102] and CAR [61,81] in a similar fashion to other NNRTI. Specifically, it has been suggested that binding of EFV to PXR inhibits PXR-co-repressor interaction and promotes PXR co-activator recruitment, thereby inducing PXR transcriptional activation [92]. Interestingly, the increase in serum ALT and AST observed in a humanized hPXR/CYP3A4 mouse model exposed to EFV (500 mg/1 kg diet for seven days) was absent in hPXR/Cyp3a-null mice, pinpointing the role of PXR activity in EFV-induced liver injury [102]. Similarly, EFV-mediated PXR activation alters hepatic lipogenic gene expression and is responsible for EFV-induced hepatic steatosis, liver damage and hypercholesterolemia in mice, as mice with liver-specific PXR deletion do not display these effects when treated with EFV [92].

In conclusion, EFV alters hepatic function, though hypersensitive reactions to EFV are far less frequent than those reported with NVP, and the mechanism of hepatic toxicity elicited by NVP and EFV are dissimilar. The effect of EFV seems to include alterations in mitochondrial function, redox state and proteostasis in the hepatic cell (Figure 3B). EFV also alters lipid metabolism, and this may be related to its ability to induce PXR.

### 3.3. Etravirine (ETR)

Current indications for ETR involve its combination with other agents in ARV treatment-experienced adult patients [12], but it is not recommended in naïve individuals due to a lack of sufficient data [41]. It is a suitable replacement for patients who are virally suppressed on other NNRTI but have developed toxicities and has become an alternative in HIV/HCV coinfected patients because of its safety and lack of interactions with anti-HCV drugs [103]. Several studies have shown the safety of ETR regarding liver function [52,104], including in HIV/HCV-coinfected patients with significant LF [40,105]. Regarding metabolic alterations, an initial study with ARV-experienced patients reported no clinically relevant changes over the 24-week study period after initiation of ETR treatment, with grade 3 or 4 changes in lipid, hepatic, and pancreatic laboratory parameters being similar to those in the placebo group [106]. Another study revealed a trend towards higher rates of grade 3 or 4 elevated triglycerides and total cholesterol but not low-density lipoprotein with ETR versus placebo [107]. Moreover, changes in lipids were less commonly observed in ETR-treated persons compared with those on EFV in the SENSE trial in a univariate analysis of changes in lipid markers from baseline to week 48 [108].

Mild serum aminotransferase elevations occur in patients on ETR-containing therapy but increases over five times the upper limit of normal values occur in only 2% to 3% of patients, and these rates are higher in patients who have hepatitis C coinfection [16,52]. The major adverse event-related reason for discontinuation of ETR is skin rash (occurring in 10–20% of patients 2–6 weeks after initiation of therapy), which can be accompanied by other signs of hypersensitivity, including immunoallergic hepatitis, whose mechanism is probably associated with the extensive metabolization of ETR in the liver via the P450 system (CYP 2C19, 3A4, and 2C9), which may generate an immunogenic metabolic intermediate. The pathogenic mechanisms of ETR´s hypersensitivity are different to those of first-generation NNRTIs.

In vitro, ETR is described as an agonist of several nuclear receptors that are abundant in the liver. Namely, mechanistic studies have shown ETR to be an activator of hPXR by an action that involves binding to the ligand-binding domain (LBD) of the receptor and recruitment of steroid receptor coactivators (SRC)-1, SRC-2, and SRC-3, thus inducing expression of the target gene CYP3A4, as determined in primary human hepatocytes [82]. ETR also triggers nuclear translocation of hCAR and increases the expression of its target gene CYP2B6; however, in the study in question, the mechanism did not appear to involve binding to the LBD or recruitment of SRC-1, SRC-2, or SRC-3 [83].

In conclusion, ETR is not considered to be particularly hepatotoxic and its effects on the liver are thought to be part of the hypersensitivity reactions that this drug can trigger.

### 3.4. Rilpivirine (RPV)

RPV is currently indicated as part of “alternative initial treatment” or “recommended initial regimens in certain clinical situations” in cART-naïve patients with HIV-RNA of <100,000 copies/mL and a CD4 count >200 cells/mm^3^, as well as in treatment-experienced patients with virological suppression and without NNRTI-resistance mutations [41,42]. Very recently, in January 2021, FDA approved the first complete long-acting injectable ARV regimen consisting of cabotegravir and RPV, as an option to replace the current ARV regimen in adults with HIV [41].

Clinically apparent hepatotoxicity due to RPV can occur, but is rare, and elevations above 5 times the upper limit of normal are uncommon, occurring in 1–4% of patients [17,39,109]. Moreover, the rate of serum aminotransferase elevations is higher in patients with HBV or HCV coinfection (~10% have values greater than 5 times the ULN). Furthermore, some studies report a slight increase in certain plasma transaminases after switching from an existing cART (mainly associated with PI) to RPV-including regimens [110,111]. RPV is considered safer than first-generation NNRTIs; for e.g., in the ECHO and THRIVE studies comparing RPV with EFV, the former was associated with a significantly lower incidence of grade 2–4 ALT and AST plasma elevations, with no serious treatment-related hepatic adverse events [109]. RPV´s hepatotoxicity is likely to be hypersensitivity-related due to the fact that RPV is extensively metabolized in the liver cytochrome P450 system (predominantly CYP3A4), which can produce toxic or immunogenic intermediates capable of triggering liver injury. So far, a few case reports of acute liver damage with RPV have been reported. In one case, the reaction was qualified as “severe allergic hepatitis”—with no rash, eosinophilia or other prominent immunoallergic features typical of the liver injury associated with NVP and EFV [112]—and, in the other, acute hepatitis was histologically determined [113].

Multiple studies have shown that RPV is safer than EFV regarding lipid abnormalities, and/or that it improves the lipid status (blood total cholesterol and triglycerides levels) of patients who have switched from EFV to RPV-containing therapies [114,115,116,117,118]. Interestingly, in the clinical trial STaR, ARV-naïve HIV patients at 48 weeks of treatment with RPV/FTC/TDF displayed the same grade 3 or 4 transaminase abnormalities as those taking EFV/FTC/TDF; however, while RPV-exposed individuals did not manifest alterations in plasma lipid levels, those receiving EFV did [114].

Regarding mechanistic studies of hepatic cell injury, evidence obtained in our laboratory has demonstrated that exposure of cultured hepatocytes to clinically relevant plasma concentrations of RPV does not affect mitochondrial function or undermine cell viability [119]. Moreover, RPV shares ETR´s ability to activate nuclear receptors CAR and PXR in vitro, and this effect occurs through different mechanisms for the two ligand-activated receptors, similarly to that observed with ETR [82,83]. One study reported that in HepG2, RPV is only slightly agonistic to PXR and less so than EFV; nevertheless, this depended on whether HepG2 cells were transfected with mPxr together with a mPxr reporter ((Cyp3a2)3-luc), or with hPXR together with hPXR reporter (CYP3A4-luc) which showed significant agonism [92]. This affirms findings in other studies of the specificity of human versus rodent PXR activation.

More recently, it has been suggested that RPV is hepatoprotective in scenarios that are unrelated to HIV infection, as it exerts a protective role as an anti-inflammatory, antisteatotic and antifibrotic agent in different animal models of chronic liver injury [120]. Specifically, these data demonstrated that RPV ameliorates LF through selective signal transducer and activator of transcription 1 (STAT1)-dependent induction of apoptosis in HSC, while promoting liver regeneration. Furthermore, the results of the retrospective analysis of the Multicenter AIDS Cohort Study (MACS) public data set carried out within the same study were in line with its preclinical results and affirmed that RPV-treated patients with HIV display better liver function than patients with HIV treated with antiretroviral regimens that do not contain RPV.

### 3.5. Doravirine (DOR)

DOR, the newest NNRTI, is approved as initial therapy, as well as for those virologically suppressed on an ARV regimen. The European AIDS Clinical Society (EACS) considers the use of DOR-containing regimens as an alternative treatment for cART-naïve patients [42], whereas the United States Department of Health and Human Services (DHHS) recommends it for certain clinical situations [41]. DOR is a promising drug; data are emerging on the potential for new combination therapy with the investigational NRTI islatravir, and as a safer alternative to various recommended first-line agents, such as INSTIs [121]. Its potential as an effective anti-HIV treatment has been highlighted, since it has the same efficacy as EFV or ritonavir-boosted darunavir and overcomes many of the described undesired effects of other antiretroviral drugs [122,123,124,125]. Moreover, DOR seems to have limited cross-resistance with other NNRTIs [126,127] and irrelevant drug–drug interaction, with the exception of strong CYP inducers or inhibitors, as DOR is metabolized by cytochrome P450 3A (CYP3A) [128,129].

Serum aminotransferase elevations have been reported in 13% of patients on DOR-containing therapy, but elevations above five times the upper limit of normal are uncommon, occurring in 1% or less of patients [18]. Dosage adjustment in patients with mild or moderate hepatic impairment is not needed, since it does not affect its pharmacokinetics [130]. DOR has not been linked to cases of acute hepatitis, acute liver failure, chronic hepatitis or VBDS. There is no information on cross sensitivity to hepatic injury between DOR and other NNRTIs, though there may be cross reactivity in the case of rash [18].

Importantly, DOR has a relatively good metabolic profile, and this may be of relevance regarding its actions (or lack of them) on the liver. It has been associated with a better lipid profile, as it only slightly diminished total cholesterol, LDL, and triglycerides in treatment-naïve adults with HIV 48 and 96 weeks after initiation of treatment, while EFV was associated with an increase in these parameters [122]. On the other hand, weight gain in treatment-naïve adults with HIV-1 was low and similar for DOR and EFV-based regimens through 96 weeks of treatment [131].

In conclusion, little is known at present about the effect of DOR on the liver, and there is no evidence of DOR causing liver damage. Nevertheless, given that the clinical use of the drug is recent, further studies are needed.

## 4. From DILI to Chronic Liver Disease

After the broad implementation of cART, the spectrum of liver disease in HIV-infected patients has shifted from AIDS-related neoplasms and direct detrimental effects of HIV infection on the liver, including elevated liver enzymes, hepatomegaly and liver steatosis, to medication-related hepatotoxicity, concomitant HCV or HBV infections, NAFLD and alcoholic disease. First-generation NNRTIs are less liver-safe than their second-generation counterparts, and multiple studies have provided insights into the mechanisms of first-generation NNRTI-based hepatotoxicity, though almost exclusively in preclinical settings. Nevertheless, a large gap exists between our understanding of the effects of NNRTIs on the liver and their contribution to the development of CLD. Given the knowledge of the pathogenesis of CLD, including processes such as mitochondrial dysfunction, oxidative stress, altered lipid metabolism, ER stress and proinflammatory signaling, it would be useful to explore the role of NNRTIs in these pathogenic phenomena, particularly regarding their ability to amplify the effects of already existing liver-damaging agents and conditions.

## 5. Conclusions and Future Perspectives

The development of cART has altered the nature of HIV disease, transforming an almost fatal illness into a chronic but apparently stable condition. Among the most prevalent complications in HIV patients are those affecting the liver; however, limited knowledge of the mechanisms of hepatotoxicity renders our understanding of the long-term potential of cART to undermine liver function insufficient. The detection of the subtle mechanisms that lead to potential drug hepatotoxicity is of key importance and remains a major challenge to clinical practice. It is of note that assessing DILI in cultured hepatocytes in vitro is often performed with exposures to higher than clinical plasma C_max_ concentrations, thus impeding clinical interpretations of mechanistic pathway changes. In addition, diminished responsiveness to drugs is a frequent artifact of the in vitro approach which is related to the presence of altered baseline conditions of the hepatocytes particularly when cancer cell lines are employed. Moreover, determining or predicting indirect and idiosyncratic mechanisms of toxicity to hepatocytes in vitro is complicated due to the lack of extrinsic mechanisms mediated by non-parenchymal cells.

DILI can arise from a variety of mechanisms that involve multiple nuclear receptors and their signaling cascades, resulting in a diverse set of cellular responses that can trigger compensatory protective actions or cell death. Though the effects of a drug can be directly mediated by the parent compound or their metabolites on hepatocytes, there can often be indirect immunological mechanisms involving the recruitment of circulating immune cells via antigen-presenting mechanisms resulting from adduct formation with reactive intermediates. First-generation NNRTIs have been associated with a number of mechanisms that can lead to liver damage, including mitochondrial toxicity and hypersensitivity. Second-generation NNRTIs seem to be liver-safe, with RPV even showing hepatoprotective properties in the context of HIV-independent scenarios of liver injury in preclinical studies, though clinical evidence of its utility is still lacking. Moreover, with currently available revolutionary new therapies for HCV, the epidemiology of liver disease in the setting of HIV infection is now shifting away from viral hepatitis towards NASH and cirrhosis, and this phenomenon needs to be evaluated in the context of cART-induced liver toxicity.

## Figures and Tables

**Table 1:** Summary of the basic pharmacological characteristics of each NNRTI. If not specified, data are obtained from European Medicines Agency (EMA) product information. OD: once daily; BD: twice a day.

NNRTI	Commercial Name	Recommended Dose (Adults)	Bioavailability	Pharmacokinetics Concentrations (μg/mL)	Main Hepatic Metabolism
Nevirapine	Viramune	200 mg orally OD for the first 14 days, followed by 200 mg BD or 400 mg OD	After oral administration: >90%	In patients taking 200 mg OD: steady state plasma C_max_ of 5.74 (5.0–7.44) and C_min_ of 3.73 (3.2–5.08) (median and range)	CYP3A4 CYP2B6
Efavirenz	Sustiva Stocrin	600 mg orally OD	After oral administration: 40–50%	In patients taking 600 mg OD: steady state plasma C_max_ of 4.072 ± 1.167 and C_min_ of 1.767 ± 1.01 (mean ± SD)	CYP2B6 CYP3A4 [11]
Etravirine	Intelence	200 mg orally BD	Absolute bioavailability unknown	In patients taking 200 mg BD: C_max_ of 0.586 (0.199–3.13) and C_min_ of 0.297 (0.075–2.71) (median and range) [12]	CYP3A4 CYP2C9 CYP2C19
Rilpivirine	Edurant Rekambys	25 mg orally OD, or long-acting intramuscular injection of 900 mg initially followed by 600 mg monthly or 900 mg every 2 months	Absolute bioavailability unknown	C_max_ (mean ± SD) in patients taking 25 mg orally OD: 0.134 ± 0.072. In volunteers injected with a loading dose of long-acting RPV of 1200 mg on day 1 was 0.140 ± 0.016, 600 mg on day 29 was 0.120 ± 0.04 and 600 mg on day 57 was 0.132 ± 0.019 [13]	CYP3A4 CYP3A5 [14]
Doravirine	Pifeltro	100 mg orally OD	After oral administration: 64%	In patients taking 100 mg OD: steady state plasma C_max_ of 0.962 (19) (geometric mean and % CV)	CYP3A4 CYP3A5 [15]
